# Socioeconomic status and family functioning influence oral health literacy among adolescents

**DOI:** 10.11606/s1518-8787.2020054001842

**Published:** 2020-03-12

**Authors:** Roanny Torres Lopes, Érick Tássio Barbosa Neves, Laio da Costa Dutra, Monalisa Cesarino Gomes, Saul Martins Paiva, Mauro Henrique Nogueira Guimarães de Abreu, Fernanda Morais Ferreira, Ana Flávia Granville-Garcia

**Affiliations:** I Universidade Estadual da Paraíba Programa de Pós-Graduação em Odontologia Campina GrandePB Brasil Universidade Estadual da Paraíba . Programa de Pós-Graduação em Odontologia . Campina Grande , PB , Brasil; II Universidade Federal de Minas Gerais Faculdade de Odontologia Departamento de Odontopediatria e Ortodontia Belo HorizonteMG Brasil Universidade Federal de Minas Gerais . Faculdade de Odontologia . Departamento de Odontopediatria e Ortodontia , Belo Horizonte , MG , Brasil; III Universidade Estadual da Paraíba Faculdade de Odontologia Departamento de Odontologia Campina GrandePB Brasil Universidade Estadual da Paraíba . Faculdade de Odontologia . Departamento de Odontologia . Campina Grande , PB , Brasil

**Keywords:** Adolescent, Self-Care, Family Relations, Health Education, Dental, Oral Health, Socioeconomic Factors

## Abstract

**OBJECTIVE:**

Evaluate socio-demographic, family and behavioral factors associated with oral health literacy (OHL) in adolescents.

**METHODS:**

Cross-sectional study conducted with adolescents aged 15 to 19 years in Campina Grande, Brazil. Parents/guardians answered a questionnaire addressing socio-demographic data. The adolescents answered validated instruments on family cohesion and adaptability (family adaptability and cohesion evaluation scale), drug use (alcohol, smoking and substance involvement screening test), type of dental service used for last appointment and OHL (Brazilian version of the Rapid Estimate of Oral Health Literacy in Dentistry). Two dentists were trained to evaluate OHL (K = 0.87–0.88). Descriptive analysis was performed, followed by Poisson regression analysis (α = 5%). A directed acyclic graph was used to select independent variables in the study.

**RESULTS:**

The following variables remained associated with better OHL: high mother’s schooling level (RR = 1.07; 95%CI: 1.03–1.12), high income (RR = 1.04; 95%CI: 1.01–1.09), white ethnicity/skin color (RR = 1.05; 95%CI: 1.01–1.10), married parents (RR = 1.04; 95%CI: 1.01–1.09), “enmeshed” family cohesion (RR = 1.21; 95%CI: 1.12–1.30), “structured” (RR = 1.06; 95%CI: 1.01–1.12) or “rigid” (RR = 1.11; 95%CI: 1.04–1.19) family adaptability, having more than five residents in the home (RR = 1.07; 95%CI: 1.01–1.14) and having used a private dental service during the last appointment (RR = 1.08; 95%CI: 1.03–1.13).

**CONCLUSION:**

Family functioning and socio-demographic factors influence the level of oral health literacy among adolescents.

## INTRODUCTION

Oral health literacy (OHL) is the ability to understand and interpret information on dental care to maintain good oral health. Thus, OHL has been associated with oral health-related behaviors and dental outcomes ^1^ . However, a direct association between oral problems and OHL has not yet been consolidated in the literature ^2^ . This association is believed to be influenced by other aspects that act as intermediary factors in the process.

The Rapid Estimate of Adult Oral Health Literacy (REALD-30) was designed as a screening tool for individuals with low OHL in different contexts. The Brazilian version (BREALD-30) has proven to be reliable, is easy to administer and has good psychometric properties for use on adults ^3^ and adolescents ^4^ in the country.

Previous studies on OHL have prioritized adults or evaluated the OHL of parents/guardians associated with oral problems in children (proxy measure) ^5^ , leaving a gap in the OHL’s literature regarding adolescents.

Adolescence is characterized by the pursuit of greater autonomy in making health-related decisions and a decrease in adult supervision ^6^ . Described as an “index age” by the World Health Organization, the age range between 15 and 19 years of age is considered a target population for epidemiological studies ^7^ . This is the first study to evaluate the influence of family functioning and social status on OHL among middle to late adolescents. Low OHL levels are believed to impact oral health behaviors and contribute to disparities in access to oral health services ^1^ . Thus, family functioning and social aspects need to be better understood to support oral health strategies aimed at this specific age group.

Inappropriate health behaviors, drug use and risk behaviors are common in adolescence. Substance use is associated with lower decision-making capacity and greater probability of inappropriate behavior, making individuals in this phase vulnerable to health problems ^8^ . Thus, substance use is believed to influence the level of OHL in adolescents.

Other important aspects that can affect OHL are the quality and structure of family relations and the type of dental service used. Appropriate family functioning is known to favor the learning process and academic performance ^9^ and may therefore be associated with OHL. Moreover, the quality of care offered at dental services and communication with a dentist varies among the different types of services ^10^ , which can influence the OHL’s level.

Among socioeconomic factors, family income has influenced the academic performance of adolescents ^11^ . As socioeconomically underprivileged adolescents may exhibit a low level of literacy, it is important to study the association between social class and OHL.

Therefore, this study aimed to evaluate associations between the level of oral health literacy and socio-demographic aspects, family characteristics and behavioral factors in a school-based representative sample of adolescents. To learn the factors associated with OHL in adolescents is important in improving health-related communication and achieving better oral health.

## METHODS

### Study design and sample

This study was conducted in Campina Grande, a center of technological and industrial development in northeast Brazil, despite its worrisome social problems ^12^ . An analytical, cross-sectional study was conducted at schools in the city with adolescents aged between 15 and 19 years, who were selected using a two-stage sampling process. First, 32 schools (16 public and 16 private) were randomly selected from a total of 131 schools in the city. Next, adolescents were randomly selected through probabilistic sampling (simple random sampling method) to compose a sample proportional to the population of each of the city’s administration districts. The sample size was calculated considering probabilistic cluster sampling for complex samples, 95% significance level, 5% acceptable margin of error, 50% prevalence rate and 1.6 factor for the design effect. The sample was then increased to compensate for a possible 20% dropout rate. Thus, the final sample comprised 769 adolescents.

### Eligibility criteria

Adolescents between the ages of 15 and 19 enrolled at public and private schools in the city were included in the study. We excluded individuals with physical, sensory, mental or behavioral limitations according to educators’ reports.

### Theoretical training and calibration exercises

OHL was measured using the BREALD-30. Two examiners were trained by an expert in the field, stablishing both inter-examiner and intra-examiner agreement ^13^ . First, theoretical-practical training with criteria for the administration of the BREALD-30 was performed, and the results were compared between examiners and expert for the determination of inter-examiner agreement using the Kappa statistic (K = 0.87–0.88). During the calibration phase, the examiners watched 15 videos of individuals with different levels of OHL and attributed scores to each case. Intra-examiner agreement was calculated by comparing the results of an initial evaluation with the repetition of the evaluation after a seven-day period (K = 0.87–0.89). The results were compared to those of the expert and discussed.

### Pilot study

A pilot study was conducted with 50 students (25 from a public school and 25 from a private school) to test the applicability of the methods and make any necessary adjustments. The results of the pilot study revealed that no changes to the proposed methods were needed. The adolescents who participated in this step were not included in the main study.

### Data collection

A socio-demographic questionnaire was sent to the parents/guardians via the adolescents to collect the following data: adolescent’s sex, self-declared ethnic background, number of residents in the home, position in the family, family income, mother’s schooling level and parent’s/guardian’s age. The parents/guardians needed to be literate and the data collection period was October 2016 to July 2017. The adolescents were also asked about type of dental service (public or private) used for their last appointment.

The Brazilian Economic Classification Criteria ^14^ were used to categorize the families into economic classes based on the education level of the head of the household and the number of consumer goods reported by the parents/guardians, such as washing machine, freezer, DVD player, personal computer, dish washer, microwave oven, refrigerators, motorcycles and automobiles (ABEP – Brazilian Association of Research Firms, 2015). Scores were attributed to each item and the sum total was used to classify the families into Class A (45 to 100 points), Class B1 (38 to 44 points), Class B2 (29 to 37 points), Class C1 (23 to 28 points), Class C2 (17 to 22 points) and Class D/E (0 to 16 points).

The validated Brazilian version of the Rapid Estimate of Adult Literacy in Dentistry (BREALD-30) was used to measure the OHL of the adolescents. This instrument is used to determine word recognition capacity. The adolescents were asked to read thirty words related to dentistry in increasing order of difficulty ^4^ . Each correctly pronounced word was awarded one point. The total was then obtained, with higher scores denoting a higher level of OHL.

The Family Adaptability and Cohesion Evaluation Scale (FACES III) developed by the University of Minnesota, whose use was validated in Brazil ^15^ , was administered to the adolescents to evaluate family functioning. FACES III comprises 20 questions to which the respondent marks one of five response options ranging from “hardly ever” (1 point) to “almost always” (5 points). The scores obtained by the sum of odd numbered questions indicate the type of family cohesion and those obtained by the sum of even numbered questions indicate the type of family adaptability ^16^ .

Family cohesion regards the emotional ties that unite family members and can be classified as “disengaged” (very low cohesion, greater distancing and independence among family members), “separated” (low to moderate cohesion, family members share some activities), “connected” (moderate to high cohesion, greater sharing of activities and greater interest in family ties) or “enmeshed” (very high cohesion, greater emotional dependence with a focus on family relations). Family adaptability regards the capacity family members have to adjust to changes in the family dynamics, being classified as “rigid” (very low adaptability, the family follows the rules of a single member and no changes occur in family roles), “structured” (low to moderate adaptability, more sharing of roles), “flexible” (moderate to high adaptability, more flexibility in terms of family control and rules) or “chaotic” (very high adaptability, no defined functions or leadership) ^17^ .

Drug use was evaluated using the validated Brazilian version of the Alcohol, Smoking and Substance Involvement Screening Test (ASSIST) developed by the World Health Organization ^18^ , comprised of eight questions that address the frequency of psychoactive drug use, difficulties in performing daily activities, difficulty interrupting drug use and concerns on the part of parents/guardians. For the present study, we considered answers to the use of legal substances (tobacco and alcohol) and illegal substances (marijuana, cocaine, stimulants, opioids, sedatives, inhalants and hallucinogens) for the previous three months.

### Statistical analysis

A directed acyclic graph (DAG) ( Figure ) was created using the DAGitty software program (version 3.0) to study the relations between the dependent (OHL) and independent variables.

**Figure:**
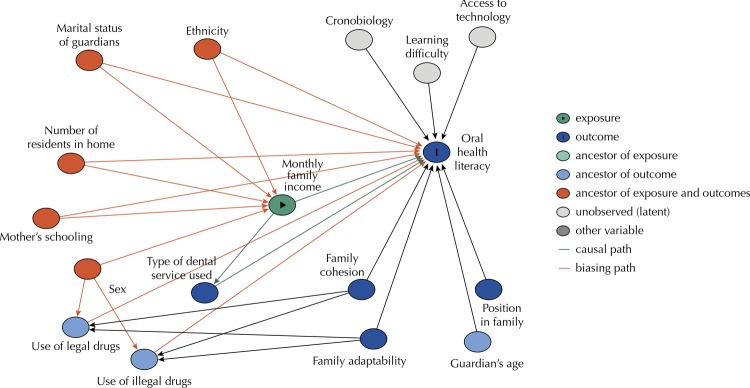
Directed Acyclic Graph (DAG) for effect of socio-demographic, family and behavioral characteristics on oral health literacy

The statistical analyses were performed with the aid of the Statistical Package for the Social Sciences (SPSS for Windows, version 22.0, SPSS Inc, Chicago, IL, USA). Descriptive analysis was performed for the characterization of the sample. Poisson regression analysis with robust variance was used to test associations between the exposure variables and outcome. For such, variables with a p-value < 0.20 in the bivariate analysis were incorporated into the multivariate analysis and those with a p-value < 0.05 in this analysis were kept in the final model. Rate ratios (RR) and 95% confidence intervals (CI) were calculated for the associations found in both the unadjusted and adjusted statistical models. Oral health literacy was used as a count outcome because cutoff points can vary significantly across populations and there is no standard categorization in the literature. Therefore, for this study, OHL as a count measure was the best way to explore differences between groups.

### Ethical aspects

This study was conducted in accordance with the Declaration of Helsinki and Resolution No. 466/2012 of the Brazilian National Board of Health regarding research involving human subjects and received approval from the Human Research Ethics Committee of the Universidade Estadual da Paraíba (certificate number: 55953516.2.1001.5187).

## RESULTS

The sample comprised 746 adolescents between 15 and 19 years of age. The response rate was 97%. As students from this age group are preparing for important university admission exams, the level of absenteeism was not high. Twenty-three adolescents were excluded for being absent for the interview after three attempts. Table 1 shows the characterization of the sample.

**Table 1:** Characterization of sample of adolescents aged 15 to 19 years

Variable	Percentiles	
Oral Health Literacy	25, 50, 75	
	17, 21, 24	
Variable	Frequency	
	n	%
Socio-demographic variables		
Sex		
Female	444	59.5
Male	302	40.5
Ethnicity/skin color		
White	211	28.3
Non-white	535	71.7
Type of school		
Public	497	66.6
Private	249	33.4
Monthly family income		
≤ US$ 240	272	51.0
>US$ 240	261	49.0
Mother’s schooling level		
< 8 years of study	299	40.3
≥ 8 years of study	443	59.7
Parent’s/Guardian’s age		
≤ 42 years	384	51.6
> 42 years	360	48.4
Number of residents in home		
≤ 5	619	83.2
≥ 6	125	16.8
Marital status of parents/guardians		
Married	403	54.1
Single, divorced, widowed	342	45.9

Family/behavioral variables		
Position in family		
Oldest child	314	42.1
Middle child	184	24.7
Youngest child	248	33.2
Use of legal drugs		
Yes	301	40.3
No	445	59.7
Use of illegal drugs		
Yes	54	7.2
No	692	92.8
Family cohesion		
Enmeshed	15	2.0
Connected	121	16.2
Separated	266	35.7
Disengaged	344	46.1
Family adaptability		
Rigid	122	16.4
Structured	216	29.0
Flexible	244	32.8
Chaotic	163	21.9
Type of dental service used		
Public	264	40.4
Private	389	59.6

After the adjustments in the multivariate analysis ( Table 2 ), mothers with more than eight years of study had, on average, 7% higher OHL scores than mothers with eight or fewer years of study. OHL scores were also higher for adolescents from families with a monthly income higher than US$ 240, those living in a home with up to five residents, those who declared white ethnicity/skin color, those whose parents were married and those who used private dental services. Adolescents categorized as having “enmeshed” family cohesion had, on average, 21% higher OHL scores than adolescents with “disengaged” family cohesion. Students with “rigid” or “structured” family adaptability respectively had 11% and 6% higher scores than those with “chaotic” family adaptability.

**Table 2:** Poisson regression for oral health literacy and socio-demographic, family and behavioral characteristics in adolescents aged 15 to 19 years

Variables*	Oral health literacy	Bivariate		Multivariate	
	Mean (SD)	Unadjusted Rate Ratio(RR)**		Adjusted Rate Ratio(RR)	
Sex					
Male	20.23 (5.30)	1.00		–	–
Female	20.43 (4.89)	1.01 (0.97–1.04)	0.597	–	–
Mother’s schooling level					
≤ 8 years of study	18.60 (5.51)	1.00		1.00	
> 8 years of study	21.50 (4.35)	1.15 (1.11–1.20)	< 0.001	1.07 (1.03–1.12)	0.001
Monthly family income					
≤ US$ 240	19.11 (5.05)	1.00		1.00	
> US$ 240	21.68 (4.63)	1.13 (1.08–1.18)	< 0.001	1.04 (1.01–1.09)	0.049
Parent’s/Guardian’s age					
≤ 42 years	20.09 (5.02)	1.00		–	–
> 42 years	20.61 (5.10)	1.02 (0.99–1.06)	0.163	–	–
Number of residents in home					
≤ 5	20.68 (4.86)	1.09 (1.03–1.15)	0.001	1.07 (1.01–1.14)	0.032
> 5	18.87 (5.69)	1.00		1.00	
Ethnicity/skin color					
White	21.54 (5.23)	1.08 (1.04–1.12)	< 0.001	1.05 (1.01–1.10)	0.010
Non-white	19.88 (4.91)	1.00		1.00	
Marital status of parents/guardians					
Married	20.97 (5.02)	1.06 (1.03–1.10)	< 0.001	1.04 (1.01–1.09)	0.040
Single/divorced/widowed	19.61 (5.02)	1.00		1.00	
Position in family					
Oldest child	20.88 (4.91)	1.02 (0.98–1.06)	0.240	–	–
Middle child	19.43 (4.71)	0.95 (0.91–1.01)	0.057	–	
Youngest child	20.36 (5.41)	1.00		–	–
Use of legal drugs					
No	20.26 (5.07)	1.00		–	–
Yes	20.50 (5.04)	1.01 (0.97–1.04)	0.526	–	–
Use of illegal drugs					
No	20.36 (5.04)	1.01 (0.93–1.08)	0.829	–	–
Yes	20.20 (5.32)	1.00		–	–
Type of dental service used					
Public	19.13 (5.06)	1.00		1.00	
Private	21.50 (4.46)	1.12 (1.08–1.16)	< 0.001	1.08 (1.03–1.13)	< 0.001
Family cohesion					
Enmeshed	24.73 (3.36)	1.26 (1.17–1.35)	< 0.001	1.21 (1.12–1.30)	< 0.001
Connected	21.07 (4.98)	1.07 (1.02–1.13)	0.004	1.01 (0.95–1.07)	0.727
Separated	20.79 (4.93)	1.06 (1.02–1.10)	0.003	1.03 (0.98–1.08)	0.153
Disengaged	19.57 (5.09)	1.00		1.00	
Family adaptability					
Rigid	21.11 (5.06)	1.09 (1.02–1.16)	0.005	1.11 (1.04–1.19)	0.001
Structured	20.81 (4.57)	1.07 (1.02–1.13)	0.007	1.06 (1.01–1.12)	0.043
Flexible	20.24 (4.98)	1.04 (0.99–1.10)	0.101	1.03 (0.97–1.10)	0.262
Chaotic	19.34 (5.63)	1.00		1.00	

* Independent variables defined using directed acyclic graph (DAG)

** Unadjusted Poisson regression for socio-demographic, family and behavioral characteristics and oral health literacy among adolescents aged 15 to 19 years

RR = rate ratio

## DISCUSSION

Adolescents with a better socioeconomic status, those who used private dental services and those from families with “enmeshed” type of cohesion and “rigid” or “structured” types of adaptability had better levels of oral health literacy. These results are important, showing, for the first time, factors related to OHL in adolescents. The present findings could contribute to devising public policies directed at this age group. We believe this is the first study to investigate factors associated with OHL in adolescents.

Important associations were found between characteristics of family relations and OHL in adolescents. The association between high family cohesion (“enmeshed” type) and better levels of OHL has also been reported in a previous study, which found that high family cohesion can influence learning levels among children ^19^ and that communication between parents and children can affect health-related behaviors in adolescents ^20^ .

The effect of family adaptability on OHL has not previously been reported. In the present study, adolescents from families with less adaptability (“rigid” and “structured” types) had higher levels of OHL. In such families, authority structure is strict, which may have favored the supervision of the adolescents and contributed to better educational results ^21^ . Consequently, less family adaptability may have favored greater access to information regarding oral health.

High mother’s schooling level has been associated with greater OHL among 12–15 years old adolescents, which may be due to the cognitive influence of this variable, as mothers with a higher educational level tend to invest more in their children’s education, which affects learning and, consequently, the ability to understand and interpret information ^22^ . This may explain the association between higher mother’s schooling level and higher levels of OHL among the adolescents in the present investigation, the first study to investigate the impact of this variable on the OHL of late adolescents.

The association between family income and OHL has also been reported in a previous study involving parents/guardians of children with dental caries ^23^ . High family income favors the availability of financial resources and contributes to greater access to dental services and communication with the dentist ^24^ , possibly contributing to better OHL.

A smaller number of residents in the home was also associated with a higher level of OHL. This finding likely indicates less division of financial resources among family members and the opportunity for greater attention directed at education in families with a smaller number of children ^25^ , possibly influencing OHL.

Having parents who are married contributed to a higher level of OHL among the adolescents. Children who live with married parents have better psychological maturity, are healthier and receive more attention to their needs, because of greater family support ^26^ . Therefore, this finding indicates that greater parental support has a positive impact on the level of OHL among adolescents.

White adolescents had better OHL compared with their non-white counterparts. A previous study found that non-white adolescents had a poorer academic performance and called attention to the occurrence of racial difference regarding the academic performance of economically underprivileged adolescents ^27^ . Such social inequalities occur in many societies, affecting the social inclusion of non-white individuals. Moreover, non-white adolescents may have to reconcile work and study due to economic hardship, which has a negative impact on learning capacity ^28^ , thereby affecting OHL.

Adolescents who used private dental services had better OHL. The high demand for care at public dental services in Brazil and the low priority given to adolescents at such services ^29^ may have a negative impact on communication regarding oral health, thereby affecting OHL’s levels among adolescents.

No association was found between drug use and OHL, contrary to expectations. A previous study found poorer academic performance and lower concentration among adolescents who took drugs ^30^ . The absence of an association in the present study likely occurred because only reports of use in the previous three months were considered, which may not represent chronic substance use. Future longitudinal studies should be conducted on the association between OHL and chronic drug use among adolescents. Moreover, since the study was conducted in a school setting, the adolescents may have felt inhibited to declare their actual consumption.

The main limitation of the present study is the cross-sectional design, which does not permit establishing the direction of the associations; longitudinal studies are needed for this purpose. However, this study offers an important indicator of factors associated with OHL in adolescents, which is an under-investigated field of study. The strengths of the study are the methodological care taken in conducting a pilot study, the training/calibration of the examiners and performing two-stage cluster sampling to obtain a representative sample. This study used broad inclusion criteria to represent the target population precisely and enhance its external validity. The results could contribute to devising public policies aimed at the promotion of oral health in adolescence. Such policies should include family evaluations at schools and health services ^15^ to assist in preventing problematic oral health behaviors among adolescents.

Brazilian adolescents with a better socioeconomic status, from families with “enmeshed” cohesion and “rigid” or “structured” adaptability, and those whose parents were married had better levels of OHL. These results underscore the importance of a favorable family environment to better oral health literacy.
